# Mental health effects of the Gangwon wildfires

**DOI:** 10.1186/s12889-022-13560-8

**Published:** 2022-06-14

**Authors:** Ji Sun Hong, So Yeon Hyun, Jung Hyun Lee, Minyoung Sim

**Affiliations:** 1 Department of Psychiatry, Chung-Ang University Gwangmyeong Hospital, Gyeonggi-do, Korea; 2 National Center for Disaster and Trauma, National Center for Mental Health, Seoul, Korea

**Keywords:** Disaster, Wildfires, Gangwon wildfires, Mental health, Psychosocial support

## Abstract

**Background:**

The April 2019 wildfires in Gangwon Province, South Korea forced the evacuation of 1500 individuals and cost more than $100 million in damages, making it the worst wildfire disaster in Korean history. The purpose of this paper was to investigate the mental health effects on survivors following the wildfires.

**Methods:**

Between April and May 2019, outreach psychological support services were delivered to people impacted by the wildfires. Post-disaster psychological responses using a checklist and the Clinical Global Impression Scale-Severity (CGI-S) were evaluated for 206 wildfires survivors. The CGI-S was administered consequently at 1, 3, and 6 months after baseline measurement.

**Results:**

Among four response categories, somatic responses (76.2%) were most frequently observed among the wildfire survivors. Specifically, insomnia (59.2%), anxiety (50%), chest tightness (34%), grief (33%), flashbacks (33%), and depression (32.5%) were reported by over 30% of the participants. The mean CGI-S scores were significantly decreased at 1 month (mean score = 1.94; SE = 0.09) compared to baseline (mean score = 2.94; SE = 0.08) and remained at the decreased level until 6 months (mean score = 1.66; SE = 0.11). However, participants with flashbacks showed significantly higher CGI-S scores compared to those without flashback at 6 months.

**Conclusions:**

Wildfire survivors have various post-disaster responses, especially somatic responses. While most participants’ mental health improved over time, a few of them may have experienced prolonged psychological distress after 6 months. Flashbacks were particularly associated with continuing distress. These results suggest that the characteristics of responses should be considered in early phase intervention and in follow-up plans for disaster survivors.

## Introduction

Major disasters, including floods, wildfires, earthquakes, and tsunamis increase the risk of physical injury or illness and cause various long- and short-term mental health issues for survivors [1–3]. Disaster-related factors can influence the psychiatric impact of the disaster, including disaster type [1]; intensity and duration of exposure [4]; and degree of disaster exposure (e.g., damage to one’s property, moving due to damage to one’s residence, personal or familial injury) [1, 5]. Moreover, victims of man-made disasters (e.g., wars, terrorism, accidents, hazardous materials exposure, explosions, or groundwater contamination) frequently experience anger, a state of suspiciousness, guilt, and self-blame [6, 7]. However, natural disasters (e.g., earthquakes, floods, hurricanes, drought, volcanoes, tornadoes, or tsunamis) mainly cause loss of property and a lack of control over one’s possessions [8–10]. Wildfires can possess the characteristics of both types of disasters depending on their cause. Specifically, if wildfires originate from natural causes, such as lightning or climate change, then they are considered as natural disasters. On the other hand, if wildfires are caused by human hazards or have an element of human intent, such as campfires being left burning, then they are considered man-made disasters. Wildfires in this study were characterized as both natural and man-made disasters because they were caused by strong winds (climatic conditions) and sparks (element of human intent) [11].

Wildfires can harm people’s mental health. Specifically, wildfire survivors commonly exhibit various physical, psychological, and cognitive reactions including nightmares, insomnia, anxiety about the recurrence of wildfires, helplessness, and re-experience or flashbacks due to overwhelming trauma experiences, such as witnessing the fire [12–14]. Studies investigating the psychiatric disorders of wildfire survivors indicate that they exhibit an increased rate of post-traumatic stress disorder (PTSD) [15, 16]. They also experience increased depression and anxiety symptoms [12, 13]; psychological distress levels [17]; and intake of alcohol, drugs, and hypnotics [18]. Moreover, significant predictors of wildfire-related psychological problems in wildfire survivors were fear for their own or their loved one’s lives, bereavement of someone lost to fire, property loss, witnessing homes being destroyed, pre-existing mental illness, low community cohesion, and recent life stressors [15, 19–21]. In some cases, wildfire-related mental health problems can persist for a long time. For example, a study on the survivors of the Ash Wednesday bushfires in Australia reported that 42 and 23% of participants met the diagnostic criteria for PTSD or depression at 1 year and at 20 months following the wildfire, respectively [22]. Additionally, residents in highly affected regions of the Black Saturday bushfires in Australia still suffered from PTSD (15.6%), depression (12.9%), severe distress (12.8%), and heavy alcohol use (24.7%) three to 4 years later [23]. Another longitudinal study conducted 5 years after the Australia bushfires showed that the rate of probable PTSD (14.7%) remained high compared to national levels (4.4%); furthermore, the rate of psychological distress including probable PTSD and depression fluctuated over time [12].

Wildfires tend to occur frequently in Korea. In the past 10 years, an average of 431 wildfires have occurred per year. Additionally, 1.2 large-scale wildfires, defined as “forest damage with an area of more than 1 km^2^ or lasting more than 24 hours,” have occurred annually [11]. More recently, on April 4, 2019, the east coast sea wildfires (the Gangwon wildfires) burned 17.57 km^2^ of land and destroyed more than 2800 buildings, forcing 1524 residents to evacuate. The estimated damage was $107.2 million, making it the worst wildfire catastrophe in Korean history [11]. On April 6, the Korean Government issued a “Declaration of a Special Disaster Zone,” requiring government intervention and support. After the evacuation, many people faced displacement or unemployment because their homes or local businesses were destroyed by the fire [11]. Importantly, although there is a large international corpus of literature on the association between wildfire experiences and mental health status, no study has systematically examined the mental health effects of wildfires in Korea. Additionally, data on Asian samples are lacking. For example, the abovementioned studies constitute representative research investigating the effects of wildfires on mental health; however, they were conducted in Australia, Greece, Canada, and the United States, with primarily Caucasian samples [12–21]. Furthermore, data on immediate psychological responses to disasters, especially those obtained from clinicians, and empirical data from community samples who received psychological support, are lacking. It is crucial to assess the effectiveness of the psychological support services provided by the central and local government. This can help provide directions for how the services should be developed and structured in the future. Therefore, we investigated the mental health impacts and recovery process of survivors of the Gangwon wildfires over 6 months. We hypothesized that wildfire survivors would experience various post-disaster responses in the phase immediately after the disaster; however, most participants’ mental health would gradually improve.

## Materials and methods

### Participants and procedures

Data were obtained from the outreach psychological support program for survivors delivered by the “Integrated Mental Health Service Team for Wildfires.” The National Center for Disaster Trauma (NCT), a Korean government institution for disaster mental health management, served as the overall supervisory body. The outreach team comprised many psychiatrists and certified mental health professionals, who visited the shelters and homes for survivors to provide counseling and education on relaxation techniques and stress management. They also conducted individual psychiatric interviews.

All survivors who received psychological support services were invited to participate in this study at the beginning of the program. A total of 315 people (age ≥ 19 years) completed the initial assessment (baseline) between April and May 2019. Following the initial assessment, 206 adults agreed to be contacted for follow-up counseling via telephone. We thusly administered the Clinical Global Impression Scale-Severity (CGI-S) at 1, 3, and 6 months after the baseline assessment. Ultimately, we analyzed the data of 206 wildfire survivors who completed follow-up evaluation to assess the impact on their mental health following the wildfires.

### Measures

#### Post-disaster psychological responses-checklist

To evaluate psychological responses to the wildfires, we administered the “Post-disaster Psychological Responses-Checklist.” This was partially modified by several specialists for use in disaster mental health based on the “various responses that may occur after a disaster” (quoted in the Committee for Disaster Behavioral Health, 2015) [24, 25].

This checklist categorizes post-disaster psychological responses into four categories: emotional, somatic, cognitive, and behavioral.

Responses for each category are as follows:

- Emotional: anxiety, grief, depression, fear, helplessness, hopelessness, anger, guilt, miserableness, shame.
- Somatic: insomnia, chest tightness, fatigue, changes in appetite, pain, indigestion, tension, nausea, hyperpnea.
- Cognitive: flashbacks, difficulty concentrating, memory decline, nightmares, poor judgment, suicidal ideation, difficulty accepting the death of a loved one.
- Behavioral: extreme confusion, caution/suspicion, isolation, alcohol abuse, avoidance/denial, violence/impulsiveness, excessive smoking, drug misuse, self-harm.

Outreach team professionals conducted face-to-face interviews with participants and asked them to provide simple yes/no answers to each post-disaster psychological response item.

#### Clinical global impression scale-severity (CGI-s)

Participants’ overall mental health severity was assessed using the CGI-S developed by Guy [26]. This is a single-item scale to evaluate the severity of symptoms interfering with overall daily life function and requiring inpatient care [27]. The CGI-S rating is based on the overall impact of the symptoms, behaviors, and functions observed by clinicians over the previous 7 days.

The clinical symptom severity of participants was rated on the following 7-point scale: 1 = *normal, no illness*; 2 = *borderline ill*; 3 = *mildly ill*; 4 = *moderately ill;* 5 = *markedly ill*; 6 = *severely ill*; 7 = *most extremely ill*.

### Statistical analysis

We conducted a frequency analysis for the psychological responses. Specifically, we conducted linear mixed models (LMM) with repeated measures to examine changes in the CGI-S scores at baseline and at 1, 3, and 6 months. LMM is a model that addresses the limitations of traditional repeated ANOVA measures, including missing data on the response variable. If one measurement is missing, then the entire case is discarded. Thus, LMM was conducted to compensate for missing values, which occurred in cases where symptoms improved and ended, one-sided contact loss occurred, or participants refused further monitoring at the follow-up observation. The LMM performed in this study was a single model in which the participant (id) and time were included as random effects and fixed effects, respectively. Subsequently, we performed post-hoc multiple comparisons with Bonferroni correction to compare the CGI-S scores between measurement times controlling the type I error rate. For responses reported by more than 30% of participants, the mean CGI-S score was compared between groups with and without each response using independent t-tests.

All data were analyzed using IBM SPSS Statics 21.0 (Chicago, IL, USA).

## Results

### Demographic characteristics

Participants’ average age was 68.72 years (SD = 12.74), and most of the sample comprised adults aged over 65 years (*n =* 129, 62.6%). More than two-thirds of the sample were women (*n* = 155, 75.2%).

### Psychological responses after wildfire

We observed somatic and emotional responses in 76.2 and 71.8% of participants (*n* = 206), respectively. This was followed by cognitive and behavioral responses in 50.0 and 16.5% of participants, respectively (Table 1). Specifically, insomnia (59.2%) and anxiety (50%) responses were reported by more than 50% of the sample. Chest tightness (34%), grief (33%), flashbacks (33%), and depression (32.5%) were also observed in more than 30% of participants (Table 1).

**Table 1:** Frequency analysis of immediate psychological responses to the wildfires (baseline) (*N* = 206)

**Somatic**	Insomnia	Chest tightness	Fatigue	Changes in appetite	Pain	Indigestion	Tension	Nausea	Hyperpnea	ㅡ	Total
	122 (59.2)	70 (34)	57 (27.7)	48 (23.3)	45 (21.8)	42 (20.4)	35 (17)	17 (8.3)	14 (6.8)	ㅡ	157 (76.2)
**Emotional**	Anxiety	Grief	Depression	Fear	Helplessness	Hopelessness	Anger	Guilt	Miserableness	Shame	Total
	103 (50)	68 (33)	67 (32.5)	61 (29.6)	50 (24.3)	35 (17)	29 (14.1)	14 (6.8)	16 (7.8)	3 (1.5)	148 (71.8)
**Cognitive**	Flashbacks	Difficulty concentrating	Memory decline	Nightmares	Poor judgment	Suicidal ideation	Denial of death	ㅡ	ㅡ	ㅡ	Total
	68 (33)	44 (21.4)	39 (18.9)	28 (13.6)	17 (8.3)	14 (6.8)	1 (0.5)	ㅡ	ㅡ	ㅡ	103 (50)
**Behavioral**	Confusion	Caution/Suspicion	Isolation	Alcohol	Avoidance/Denial	Violence/Impulsiveness	Smoking	Drug use	Self-harm	ㅡ	Total
	11 (5.3)	10 (4.9)	9 (4.4)	8 (3.9)	6(2.9)	2 (1)	2 (1)	1 (0.5)	ㅡ	ㅡ	34 (16.5)

### Difference in the severity of mental health according to psychological responses

**Fig. 1:**
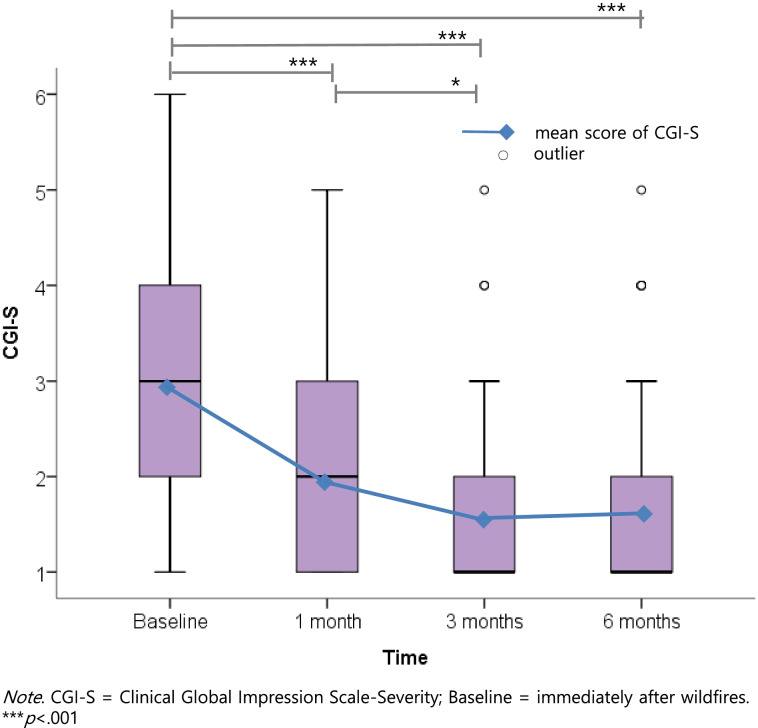
Changes in the Clinical Global Impression Scale scores over time. *Note*. CGI-S = Clinical Global Impression Scale-Severity; Baseline = immediately after wildfires. ***p < .001

The mean CGI-S score was 2.94 at baseline (*SE* = 0.08). This decreased to 1.94 (*SE* = 0.09) at 1 month, 1.62 (*SE* = 0.10) at 3 months, and 1.66 (*SE* = 0.11) at 6 months (*F* = 74.458, *p* < .001). Table 2 presents the relations between measurement times and the CGI-S. Post-hoc multiple comparisons with Bonferroni correction for CGI-S score differences showed that CGI-S scores were significantly lower at 1, 3, and 6 months compared to baseline (*p* < .001, respectively), and at 3 months compared to 1 month (*p* < .05). However, there were no statistically significant differences between 3 and 6 months. The changes in CGI-S over time are presented in Fig. 1.

**Table 2:** Relations between measurement times and the Clinical Global Impression Scale (*N* = 206)

Times	b	S.E.	df	t	***p***-value	95% CI
**(Intercept)**	1.657	.109	500	15.226	< .001***	1.443 ~ 1.871
**Baseline**	1.285	.113	355	11.353	< .001***	1.063 ~ 1.508
**1 month**	.279	.119	341	2.355	.019*	0.046 ~ 0.513
**3 months**	−.036	.122	329	−.291	.771	−.276 ~ .205
**AIC**	1475.808					

*b* Standardized Regression Coefficient, *S.E.* Standard Error, *df* Degree of freedom, *CI* Confidence Interval, *AIC* Akaike Information Criterion; * *p* < .05, *** *p* < .001

**Fig. 2:**
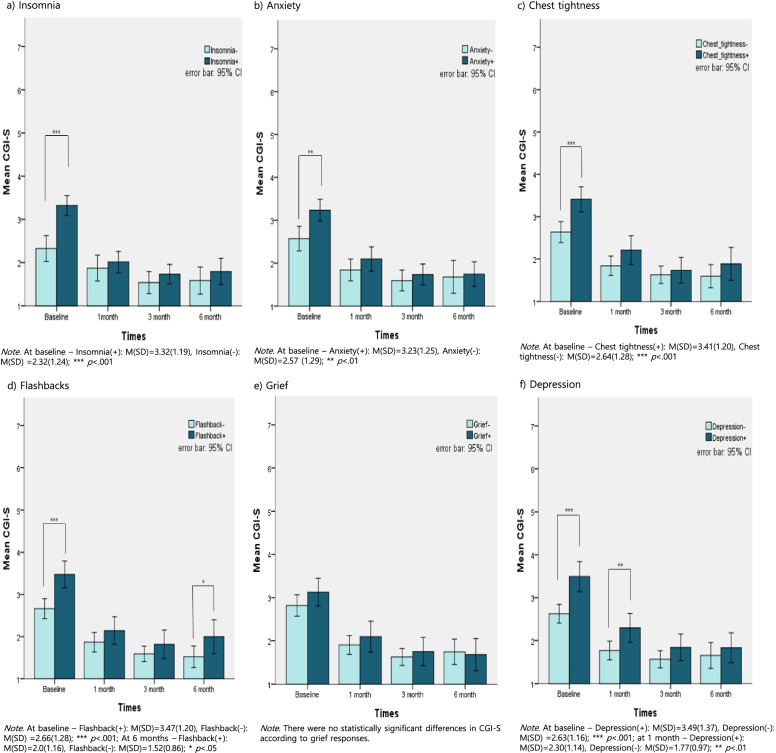
Difference in CGI-S according to psychological responses. Abbreviations: SD=Standard Deviation; CGI-S=Clinical Global Impression Scale-Severity; Baseline = immediately after wildfires

As shown in Fig. 2, the CGI-S score at baseline was higher in each group with responses compared to those without responses: insomnia (*t*(172) = 5.303, *p* < .001), anxiety (*t*(171) = 3.438, *p* < .01), chest tightness (*t*(171) = 3.943, *p* < .001), flashbacks (*t*(170) = 3.997, *p* < .001), and depression (*t*(171) = 4.388, *p* < .001).

Moreover, the mean CGI-S score at 1 month was higher in the group with depression than in the group without depression. The mean CGI-S score at 6 months was higher in the group with flashbacks compared to the group without flashbacks {*t*(123) = 2.767, *p* < .01, *t*(79) = 2.126, *p* < .05, respectively}.

## Discussion

This study investigated the mental health effects of a wildfire on affected residents in South Korea. Over 70% of the study population reported at least one of the somatic, emotional, cognitive, and behavioral stress responses immediately after the disaster experience. Most participants in our study were primary survivors who were directly exposed to traumatic stressors, such as witnessing the fire or incurring property damage. Such exposure levels are related to a high rate of psychological discomfort, which is consistent with previous studies demonstrating that mental health effects are associated with directly witnessing a fire or having one’s home destroyed [28–31].

Regarding the changes in CGI-S scores over time, the mean score decreased within 1 month after the disaster, which was maintained at 6 months. Our observation is notable in the context of previous studies. Specifically, regarding the Australian Ash Wednesday, Australian Black Saturday, and the Blue Mountain bushfires, most of the affected people eventually coped with the adversity; moreover, few people experienced probable PTSD, depression, or psychological distress [20, 22, 32–38]. However, it is unknown whether the improved CGI-S scores in the current study occurred naturally or due to the psychological support provided, since data were obtained from the group who received psychological support. Additionally, the follow-up period was only 6 months.

Among the four categories of responses, somatic responses were most frequently observed in the wildfire victims. This is in line with the finding showing that somatization is frequent in wildfire victims [13]. Acute traumatic stress is known to activate the sympathetic nervous system and evoke a neuroendocrine stress response, which are subsequently associated with post-traumatic somatic symptoms [39–41]. We considered that the socio-demographic characteristics of our participants, such as having a high proportion of older adults and women, may have partially influenced the results. Older adults and women are not only regarded as vulnerable populations regarding their psychological responses following disasters [42, 43], but also tend to complain of somatic symptoms more frequently [42, 44]. Depression, anxiety, and stress reactions are often expressed as somatic symptoms, especially in older adults [45]. In addition, the tendency to emphasize somatic symptoms when suffering psychological distress has been frequently reported in samples from East Asian cultural contexts, including Korea and China [46].

Furthermore, many participants reported experiencing vivid flashback responses in the current study. For example, they said, “The embers still fly around before my eyes” or “The embers are chasing me.” Notably, regarding flashback responses, the mean CGI-S score at 6 months after the wildfire was higher in the group which experienced flashbacks relative to the group which did not experience flashbacks. A previous longitudinal study investigating the alterations in the network structure of PTSD symptoms found that the re-experience cluster including flashbacks and distressing reminders played crucial roles until 6 months. Thus, re-experience symptoms may play a key role in the evolution and persistence of PTSD [47]. These results were consistent with other studies indicating that early re-experience symptoms predict the development of PTSD [48, 49].

In our study, 33.0% of participants reported grief responses. The wildfires destroyed their houses and households, and the survivors grieved the loss of their meaningful possessions. This suggests that survivors could experience a serious mourning reaction, not only to loss of life, but also to property. Notably, anger was reported at a low level (14.1%) compared to studies in which anger was a frequent and important mediator of psychopathology in man-made disasters [50–53]. Even though wildfires are considered as man-made disasters under Korean law, a survivor’s response might vary depending on the cause of the wildfire. In the 2019 Gangwon wildfires, an electrical short was identified as the origin, and the rapid spread was attributed to climatic and topographical characteristics [11]. Compared to previous wildfires which were mainly man-made, it was difficult to place blame for this wildfire as it was heavily influenced by natural factors.

Our findings highlight the necessity of long-term policies and intervention programs to care for individuals who are affected by disasters and experience mental health problems, as well as the need for a community-expanded approach. Consistent with this, the “Integrated mental health service team” have provided ongoing mental health programs for survivors. This includes education for community residents, long-term follow-up counseling and a psychiatric institution referral if needed, and group therapy based on stabilization and cognitive-behavioral techniques. Considering our findings regarding somatic responses and flashbacks, we suggest that body-based stabilization techniques may be more effective than cognitive approaches. Further studies are necessary to compare the effectiveness of body-based stabilization versus cognitive intervention and/or investigate the long-term effect of community-based mental health interventions on the mental health impacts of the wildfires.

### Limitations

This study had several limitations. First, variables such as demographic data, factors related to disaster experience, pre-trauma history of mental disorders, and having a social support system could not be sufficiently evaluated. Additionally, the CGI-S was the only objective measurement used in this study due to constraints in the research conditions. The primary purpose of the mental health support team was not to conduct rigorous research, but to provide optimal mental health service. Therefore, it was difficult to thoroughly design the study or gather extensive data. Previous findings indicate that a pre-disaster history of mental disorders, greater incident exposure to disaster, lack of social support, or experiencing an extra socioeconomic stressor are significant predictors for developing psychological distress after disasters [54–58]. Considering this, shortage of such data could be a major limitation of our study. However, despite these limitations in data collection, the CGI-S scales constitute an easily understood and practical measurement tool that can be readily managed by a clinician in a practice setting [27]. Second, clinical measures were based on a simple confirmation (yes or no) of each response. Because the survey methodology did not use structured clinical interviews, no formal diagnosis was possible, and our analysis is based solely on the manifestation of each response. Third, the participants in this study were not fully representative of all Gangwon wildfire survivors because only people who received mental health services were invited to participate in this study. People who are more severely affected by a disaster are more likely to seek counseling, which might contribute to an elevated measure of post-disaster psychological distress. Therefore, caution is needed when generalizing these findings. In addition, the average age of the sample was 68.72 years, which also limits the generalization of interpretation. The proportion of older adults aged 65 years and over in Gangwon Province is 19.1%, which is 14.9% higher than the rest of the nation [59]; thus, this limitation was difficult to avoid. Finally, our study lacked a control population, which is significant to determine the comparative effect of the disaster on the affected population.

Despite these limitations, our study is the first to investigate the mental health impacts of wildfires in Korean history. Knowledge from this study could inform policymakers when planning supportive programs to alleviate the mental health impacts of natural disasters.

## Conclusion

The present findings highlight several significant outcomes. First, even though many participants experienced significant psychological distress immediately after the disaster, most seemed to recover over time. Second, despite the general trend of resilience, a significant proportion of participants presented with prolonged psychological distress. Specifically, flashback responses could be a predictor of long-term psychopathology. Finally, an adequate public mental health service system is needed for survivors affected by disasters. Consequently, this study will help build more empirically informed evidence regarding how survivors’ mental health is influenced by disasters and elucidate the necessity of mental health support and programs for disaster survivors.

## Data Availability

The datasets generated and/or analyzed during the current study are not publicly available due to confidentiality; however, data is accessible from the corresponding author on reasonable request.

## Abbreviations

PTSD: Post-traumatic stress disorder
NCT: National Centre for Disaster Trauma
CGI-S: Clinical Global Impression Scale-Severity
LMM: Linear mixed models
